# Obituary – Prof Brian E. Leonard (1936–2023)

**DOI:** 10.1093/ijnp/pyae012

**Published:** 2024-02-19

**Authors:** Timothy G Dinan, John F Cryan

**Affiliations:** University College Cork, Cork, Ireland; University College Cork, Cork, Ireland


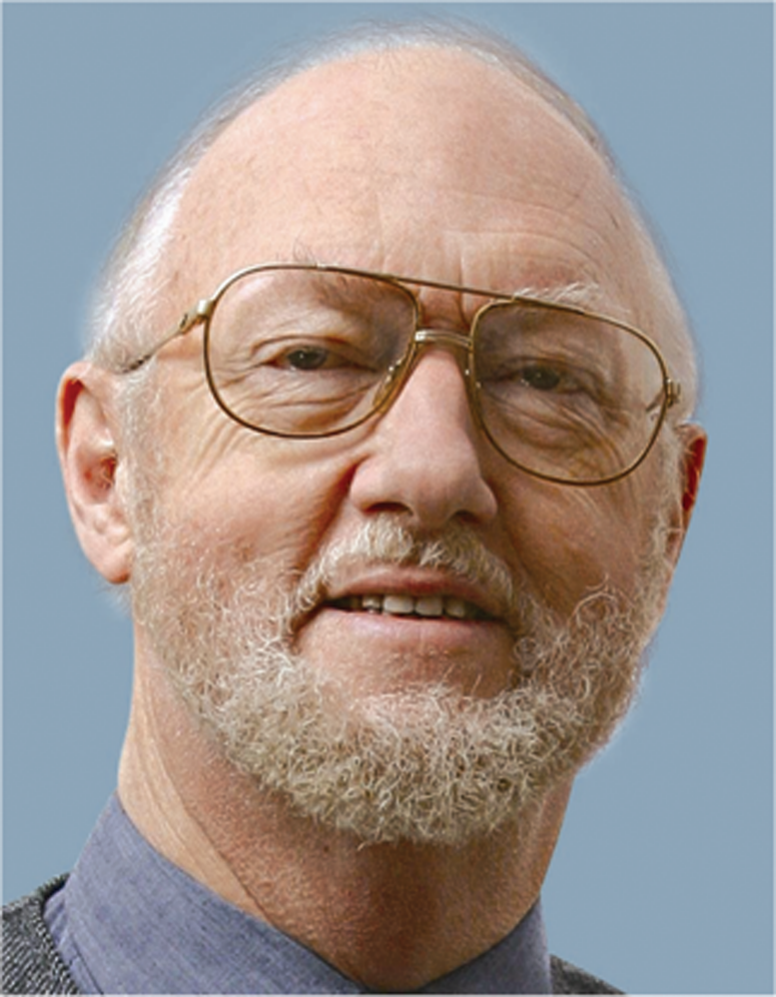


It is sad to announce the passing of Professor Brian E. Leonard, who died peacefully surrounded by his wife and daughters on December 27, 2023, at the age of 87 years. His passing is an enormous loss not just to family and friends but also to the field of psychopharmacology, where his contribution was incalculable. He was Emeritus Professor of Pharmacology at University of Galway, Ireland. Appointed as Foundation Chair of Pharmacology in 1974, his contribution was immense. He supervised more than 40 PhD and many MSc and MD students during his tenure. Many of these are now in key academic posts internationally. After his retirement, he spent several years as Visiting Professor of Pharmacology in Maastricht and held an Honorary Professorship at Ludwig Maximillian University, Munich.

Brian was a prolific publisher with a phenomenal research output of more than 450 papers across the field of psychopharmacology. His work has had major translational benefits in clinical psychiatry. For many years he worked on the pharmacology of depression and was perhaps best known for the development and characterization of animal models (most notably the olfactory bulbectomy model) and understanding the pharmacological actions of antidepressants, anxiolytics, and antipsychotic medications. In recent times, he focused on immune aspects of mood disorders and was a pioneer in the field. His initial papers in this area are of seminal importance, providing a platform for much of the current research and opening the opportunity for pharmacological interventions in depression that target the immune system. He wrote and edited numerous books, including *Fundamentals of Psychopharmacology*, which became a must-read text for trainee psychiatrists and psychopharmacologists.

Although British by birth, he very much identified with his adopted country of Ireland. He started his career at the University of Birmingham, UK, where he received a BSc and PhD in medical biochemistry in 1959 and 1962, respectively. This was followed by a lectureship at the University of Nottingham, UK, from 1962 to 1968. Next he spent very productive years in the pharmaceutical industry, first with ICI Ltd in Alderley Park until 1971 and then in 1974 he joined Organon Laboratories in the Netherlands as the Head of Neurochemistry.

He was regarded by all as a wonderful lecturer, and his impact on trainee psychiatrists and scientists in Ireland and further afield was enormous. He never spared himself and would travel anywhere to teach trainees, enthusing them with his passion for psychopharmacology. Undoubtedly, many psychiatric trainees were profoundly influenced by him and chose a path in clinical research as a result.

An enthusiastic advocate for the activities of the International College of Neuropsychopharmacology (CINP), he was president between 2004 and 2006. During his tenure, CINP membership increased substantially, especially with members from low-income countries, and the annual meetings reflected this more diverse membership. He was also Chair of the Local Organising Committee for a very successful CINP meeting in Glasgow in 1998. He was President of the British Association of Psychopharmacology between 1988 and 1990 and received that organization’s Lifetime Achievement Award in 2008.

Brian was completely dedicated to global education throughout his career and most notably in his role as chair and member of the CINP educational committee from 2006 to 2018. He was actively involved in World Health Organization training programs in low-income countries. He would probably regard this as his major contribution. In Zimbabwe, he frequently lectured in the 1980s and 1990s as well as in other parts of Africa. He often travelled to Zimbabwe with his wife, Helga. His energy levels at this stage knew no bounds, and he would frequently teach for eight hours each day to psychiatric trainees and psychiatric nurses. He felt strongly that the nurses played a fundamental role in delivering care in regions where there was no access to psychiatrists. When he was there, apart from teaching, he indulged his other pastime, collecting butterflies. After the Islamic revolution, he was one of the first to lecture in Iran. Not that he had any great sympathy for the revolution, but he felt ordinary doctors and their patients should not be isolated from global developments in pharmacology. He was friendly in his interactions with all and passionate about social causes.

Why did he concentrate his career on the pharmacology of psychiatric disease? He did so because, in his view, mental illnesses were the worst of all. He was adamant in that regard. Brian had a very extensive network of collaborators and contacts all over the globe, and he often called upon them to place some of his graduate students for extended stays. It is worth noting that there was very limited government research funding in Ireland at this time, which gives further testimony to his resourcefulness in training so many. He helped to galvanize an Irish research ecosystem in neuroscience, and in recognition he was awarded the first Distinguished Investigator Award from Neuroscience Ireland in 2011. Among the many other honors and awards bestowed on him were election as a member of the Royal Irish Academy in 1983; the Arvid Carlsson medal for education from the CINP in 2012; and the Kraepelin-Alzheimer medal for research and education, Munich University, 2012. He served on the editorial board of 6 international psychopharmacology journals and was editor-in-chief of the journal *Human Psychopharmacology* from 1995 to 2000.

Brian never fully retired. He was academically active to the very end. However, in his semi-retirement, he spent more time with his beloved wife, Helga, and travelled regularly to the UK to visit his two daughters, Ingrid and Heidi, as well as his grandchildren.

We will remember Brian Leonard as a wonderful personality and a great friend and mentor.

